# Effect of Ultrasonic Extraction on Production and Structural Changes of C-Phycocyanin from Marine *Spirulina maxima*

**DOI:** 10.3390/ijms19010220

**Published:** 2018-01-11

**Authors:** Woon Yong Choi, Hyeon Yong Lee

**Affiliations:** 1 Department of Medical Biomaterials Engineering, Kangwon National University, Chuncheon 200-701, Korea; cwy1012@hanmail.net; 2 Department of Food Science and Engineering, Seowon University, Cheongju 361-742, Korea

**Keywords:** *Spirulina maxima*, phycocyanin, ultrasonification process

## Abstract

This work first showed that very high amounts of phycocyanins, such as 11.3 mg/mL C-phycocyanin (C-PC), 3.1 mg/mL allophycocyanin (APC), and 0.8 mg/mL phycoerythrin (PE), can be obtained using an ultrasonic extraction process (UE) with a 60 kHz frequency and 3 h of process time at 25 °C, without any other pretreatments. These yields were higher than those from most conventional water extractions at 4 °C for 24 h (Control condition) or at 25 °C for 24 h (WE), namely, 9.8 and 5.7 mg/mL C-PC, 2.3 and 1.2 mg/mL APC, and 0.7 and 0.3 mg/mL PE, respectively. These yields were also shown to be even higher than yields from other reported data. Structural changes in C-PC in the extracts were also found for the first time, according to extraction conditions, showing that the total concentration of C-PC and of the α-subunit of C-PC in the UE were much higher than in the WE, with little difference in the amount of β-subunit of C-PC in the UE or WE. It was also shown that the structural changes in C-PC in the WE decreased both antioxidant and anti-inflammation activities—29.83% vs. 32.09% of α,α-diphenyl-β-picrylhydrazyl (DPPH) scavenging activity and 8.21 vs. 7.25 µM of NO production for the WE and UE, respectively—while the UE, with similar patterns to standard C-PC, showed very high biological effects, which may suggest that the biologically active part is the α-subunit of C-PC, not the β-subunit.

## 1. Introduction

Since ancient times, *Spirulina* has been considered a perfect food that has been consumed by humans because it contains high amounts of good quality protein, lipids, essential minerals, and biologically active substances such as phycocyanin, beta-carotene, chlorophyll, and gama-linoleic acid (GLA) [1,2,3]. *Spirulina* is known to be the most phycocyanin-rich natural resource on earth [3]. Phycocyanins from *Spirulina* are mostly consumed as natural edible and functional colorant due to their brilliant blue color with excellent antioxidant, anti-cancer, and anti-inflammatory activities [4,5,6]. In some applications, phycocyanins have been used as bioactive substances and not limited to use as dyes alone. Among other cyanobacteria and red algae, *Spirulina* contains the highest concentrations of phycocyanin and is a major form of soluble phycobiliproteins (ca. 20 wt % of phycobiliproteins), which can be divided into three types: C-phycocyanin (C-PC), phycoerythrin (PE), and allophycocyanin (APC) [7]. Phycocyanin is a complex biliprotein of α,β-subunit polypeptides, to which one or more linear tetrapyrrole chromophores are covalently attached and has a maximum absorbance at 610–620 nm [8]. However, although phycocyanins have many good biological activities, as already mentioned in the above [4,5,6], they have limited uses because they are very sensitive to changes in light, temperature, pH, and extraction/purification solvents, which severely reduces their extraction yield and purity [9]. This sensitivity is a major factor in its high cost and decreases its biological functions during production, especially during purification steps. Many processes have been studied to extract large quantities of intact forms of phycocyanins from *Spirulina*, such as freezing shock, lysozyme digestion, glass bead extraction, and extraction with various solvents [9,10]. Unfortunately, most have not shown good results in terms of high production yield, good stability, or high efficacy, even though some processes require large facilities and equipment and longer processing times. In general, temperatures below room temperature are considered necessary for extracting C-phycocyanin from *Spirulina*, and efficient methodologies of breaking the cell walls are also needed. Thus, ultrasonication extraction is a promising method for treating *Spirulina*, as low temperatures can be maintained; however, to date, most ultrasonication extraction processes have been performed at fixed frequencies with one input power, even though there are differences in transferring input energy into cell walls according to different input frequencies due to the special characteristics of ultrasonic generation within extraction solvents [11,12]. Therefore, in this work, the effect of ultrasonic frequency when employing simple ultrasonication extraction to obtain high amounts of intact C-phycocyanin at room temperature was observed. A lower temperature would increase production costs. The effect of the extraction condition on the integrity of the α,β-complexity of C-phycocyanin was also evaluated by comparing its biological activities to those from conventional cold water extraction processes.

## 2. Results

Table 1 shows the amounts of three major protein pigments in *Spirulina* extracts, such as C-PC, APC, and PE, from various extraction conditions with ultrasonication. 

**Figure 1:**
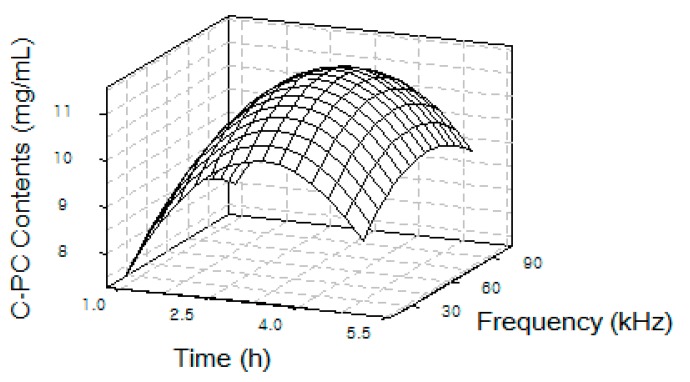
3-D contour plot of C-PC yields from *S. maxima* extract as a function of ultrasonic frequency and process time.

As shown in Table 1, the Control condition was set to 4 °C for 24 h of extraction since this condition has been most employed for obtaining C-PC from *Spirulina* so far [10]. The water extraction at 25 °C for 24 h (WE) was also designed to compare it with results from ultrasonic extraction at 25 °C to show that ultrasonic extraction increases C-PC yields. To find the optimal condition for extracting the most C-PC via ultrasonication, Figure 1 illustrates a 3-D contour plot correlating input frequency and process time to C-PC amounts isolated under various extraction conditions, as shown in Table 1. From this plot, a maximal amount of 11.3 mg/mL C-PC was obtained under ultrasonication extraction at a 60 kHz frequency and 3 h of process time at room temperature. 

Of course, a cold temperature (e.g., 4 °C) would maintain a more stable condition for extracting phycocyanins from *Spirulina*; however, in considering scale-up and economic feasibility, the cost of maintaining a lower temperature is undesirable. Therefore, for determining an optimal extraction condition, the extraction temperature was fixed to a room temperature such as 25 °C, which was also used in the 24 h water extraction (WE) for comparison. Then, various frequencies and shorter process times were applied to measure the amounts of C-PC in the extracts from each experiment. Under this optimal condition, C-PC with the highest concentration of 11.3 mg/mL was extracted, much higher than 5.7 mg/mL from the water extraction at 25 °C for 24 h.

**Figure 2:**
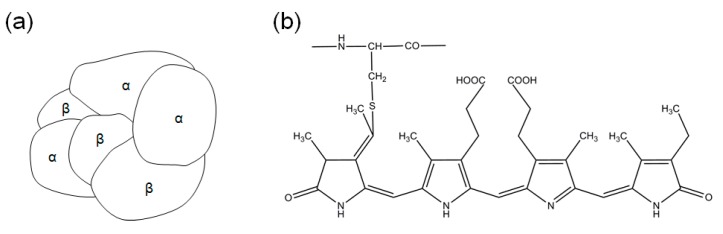
Structural diagram of C-PC (α,β) 3-trimer (**a**) and phycocyanobilin (**b**).

**Figure 3:**
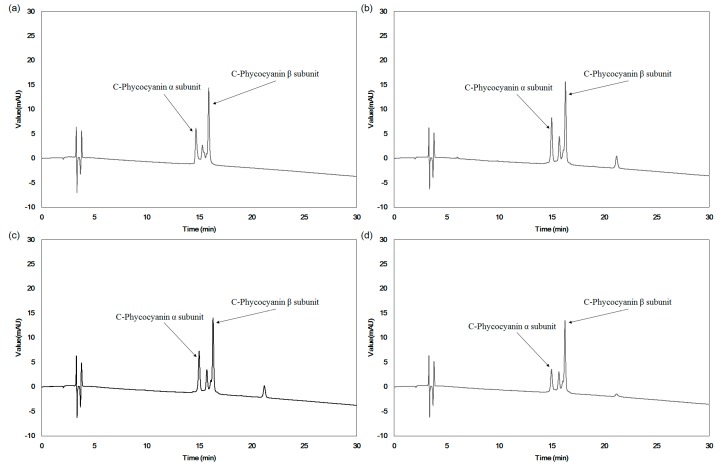
HPLC chromatograms of (**a**) pure C-Phycocyanin; (**b**) *S. maxima* extract from optimal extraction process by ultrasonication (UE); (**c**) *S. maxima* water extraction at 4 °C for 24 h (Control); (**d**) *S. maxima* water extraction at 25 °C for 24 h (WE).

Since the C-PC present in *Spirulina* consists of α- and β-subunits, as shown in Figure 2 and similar structure was also reported elsewhere [8], HPLC analysis confirmed changes in α- and β-subunits with various extraction processes. Figure 3 shows HPLC chromatograms of C-PC in different extraction conditions: a C-PC standard (a), an optimal ultrasonication extraction condition (b), water extraction as a control (c), and water extraction at 25 °C for 24 h (d). For the HPLC peaks of the C-PC standard, two major peaks were clearly observed as the α- and β-subunits of C-PC, as also reported in other works [13,14].

Two major peaks corresponding to α- and β-subunits were also shown for C-PC in the control extract (a) and optimized extract (b). However, the peak heights for the α- and β-subunits in each extract were different, indicating that the α- and β-subunits of C-PC in the extracts were different, even though the total amount of C-PC in the extracts were the same. Specifically, the α-subunit of the extract from water extraction (d) was greatly reduced, while the β-subunit was nearly unchanged compared to the α- and β-subunit peak of (b). Interestingly, the peak pattern of (b), the C-PC from the optimal ultrasonication extraction at 25 °C, was more similar to that of the standard C-PC than from water extraction at 25 °C (d). Therefore, the amount of C-PC in the extract greatly decreased and the structure of C-PC changed under harsh extraction conditions; this is a novel finding. 

**Figure 4:**
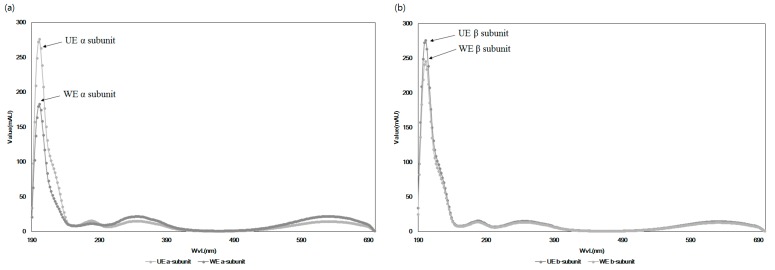
Absorption spectra of C-phycocyanin obtained from *S. maxima* using different processes. (**a**) C-phycocyanin α-subunit, (**b**) C-phycocyanin β-subunit. WE: *S. maxima* water extraction at 25 °C for 24 h, UE: *S. maxima* extract from optimal extraction process by ultrasonication.

**Figure 5:**
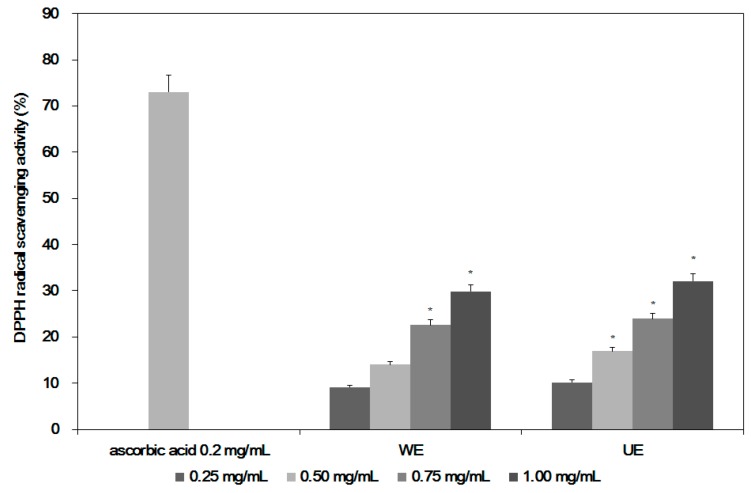
DPPH radical scavenging activity of *Spirulina maxima* extracts using different processes. WE: *S. maxima* water extraction at 25 °C for 24 h. UE: *S. maxima* extract from optimal extraction process by ultrasonication. * Significant difference, *p* < 0.05.

**Figure 6:**
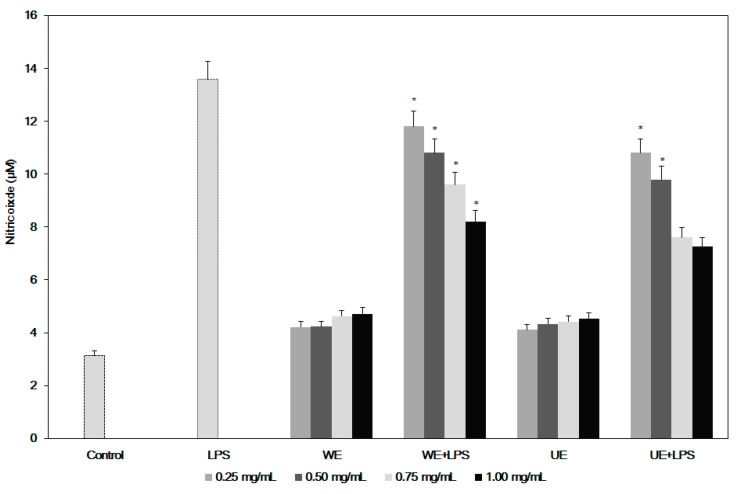
Nitric oxide content of *S. maxima* extracts by various processes. WE: *S. maxima* water extraction at 25 °C for 24 h. UE: *S. maxima* extract from optimal extraction process by ultrasonication. * Significant difference, *p* < 0.05.

This hypothesis was confirmed by comparing the UV spectra of extracts from the optimal condition and water extraction with the C-PC standard in Figure 4. Major peaks in the absorbance representing α- and β-subunits in Figure 4a,b, respectively, were also very similar to the standard, while the patterns of changing the heights of the absorbance of the WE somewhat differed from UE, with a large reduction in the α-subunit compared to WE and UE. This structural change in C-PC could reflect biological efficacy and the amounts of total phycocyanins (C-PC, APC, and PE) in the extracts from two different extracts. To confirm this hypothesis, Figure 5 and Figure 6 compared the biological effects of the extracts from each extraction condition. 

Table 2 shows the cell cytotoxicity of the extracts from two processes against macrophages (RAW 264.7) according to treatment concentrations. In general, both extracts did not show severe cytotoxicity, at 14.1% and 15.5% for UE from optimal ultrasonic extraction and WE from water extraction, respectively, even at the highest concentrations tested (1.0 mg/mL). The optimal UE extraction appeared less cytotoxic than WE even though the overall cytotoxicity of both extracts did not differ by much. 

Figure 5 shows the antioxidant activities of both extracts (UE and WE). Since C-PC is known to have antioxidant activities [5,15], the extract from an optimal condition (UE) showed better antioxidant activities, at 32.1% compared to 29.8% for WE at the highest concentration of 1.0 mg/mL, because the UE contained higher amounts of C-PC, with higher peaks for α- and β-subunits than in WE. The antioxidant activities of the UE were also higher than in other reports [16,17], possibly due to higher content of C-PC in the extract (UE) than from other processes. In Figure 5, the better antioxidant activities of C-PC from UE are shown, so good anti-inflammation activities of the extracts are expected. In addition to antioxidant activities, *Spirulina* extracts are also known to have immune-modulatory activities [5,15].

Figure 6 presents the nitric oxide (NO) production from macrophages in treating various concentrations of samples with and without lipopolysaccharide (LPS), since nitric oxide synthase (NOS) in macrophages was induced by LPS, and the production of NO was sequentially increased [18]. In treating the highest amounts of WE with LPS, 8.21 μM NO was produced, compared to 7.25 μM with UE containing high amounts of C-PC, while 13.60 μM NO was produced with LPS alone. In treating macrophages with UE or WE, only 4.71 μM NO was produced, similar to the case of the control without sample treatment. 

## 3. Discussion

As expected, under most extraction conditions, C-PC was obtained in the highest amounts, followed by APC, and the amount of PE was lowest, similar to other reported results for these pigments [19]. The amounts of PE in the extracts were negligible in most cases, similar to other reports finding no PE in *Spirulina* [19]. The C-PC yield from simple ultrasonication extraction at room temperature was the highest concentration among all tested extraction processes, e.g., 1.94 mg/mL C-PC from enzymatic treatment with water extraction [20] and 3.73 mg/mL from water extraction at 30 °C for 24 h [21]. This is the first report showing that simple ultrasonication under proper extraction conditions can produce very high amounts of intact phycocyanins without other pretreatment and/or additional extraction solvents. This may indicate that ultrasonication extraction could extract more intact C-PC than water extraction due to the shorter process time at temperatures higher than 4 °C, since C-PC is extremely sensitive to environmental temperature [11,12]. This also indicates that UE containing high amounts of C-PC has better immune modulatory activities than WE with less C-PC since in general not so many biologically active substances can be extracted at such a low temperature. Therefore, the results in this work clearly confirmed that extracts containing high amounts of intact forms of C-PC had better antioxidant and immune activation activities than WE, which had a low amount of C-PC. It also suggests a primitive hypothesis that the active portion of C-PC is the α-subunit and not the β-subunit, as the extracts that showed higher biological activities contained higher amounts of C-PC with relatively larger α-subunits and fewer β-subunits. The enhancement of its biological activities could be considered to be mainly due to C-PC, since the amounts of other bioactive substances in the extracts were very low compared to the C-PC.

## 4. Materials and Methods 

### 4.1. Materials and Preparation of the Samples

Dried marine micro alga, *Spirulina maxima* was provided by the Korea Institute of Oceanology Science and Technology (KIOST Jeju Center, Jeju, Korea), which was grown with volcanic sea water in Jeju Island, Korea, and harvested and sun-dried in 2017. All solvents and reagents used in the experiments were of analytical grade and purchased from Sigma-Aldrich (St. Louis, MO, USA). Standard C-phycocyanin (P2172) was also purchased from Sigma-Aldrich. For ultrasonic extraction, 10 g of *Spirulina* powder was added to 500 mL of distilled water and placed in an ultrasonication extractor (AUG-R3-900, ASIA ULTRASONIC, Gyeonggi-do, Korea) equipped with a multi-controller at variable frequency. At a 25 °C constant temperature, samples were extracted with 20–100 kHz frequency at 120 W power and 1–5 h extraction times (UE). The extract was filtered through a 20 μm filter paper (Whatman, Fairfield, CT, USA) and condensed using a rotary vacuum evaporator (Eyela, Tokyo, Japan). The extracts were subsequently freeze-dried using a lyophilizer (IlShin Co., Seoul, Korea) before use. For the control, 50 g of *Spirulina* powder with 500 mL of distilled water was also extracted in a flask by agitation at 50 rpm at 4 °C for 24 h (Control) and 25 °C for 24 h (WE). The extract was also freeze-dried using the same procedures used for other samples. 

### 4.2. Measurement of C-Phycocyanin, Allophycocyanin, and Phycoerythrin Content in the Extracts

The quantity of three major phycobiliproteins in *S. maxima* extracts—C-phycocyanin (C-PC), allophycocyanin (APC), and phycoerythrin (PE)—were measured using the following method [7,10]: First, 100 mg of extracts were placed in a 15 mL tube and mixed with 10 mL of 0.1 M phosphate buffer (pH 7.0) for 1 min. The supernatants from centrifugation at 1329× *g* for 5 min were collected, and absorbance was measured at 562 nm, 615 nm, and 652 nm, respectively. The amounts of C-PC, APC, and PE were calculated using the following equations [7,10]:

[C-PC (mg/mL)] = (O.D 615 − 0.474 (O.D 652))/5.34(1)

[APC (mg/mL)] = (O.D 652 − 0.208 (O.D 615))/5.09(2)

[PE (mg/mL)] = (O.D 562 − 2.41 (C-PC) − 0.849 (APC))/9.62.(3)

After measuring the contents of C-PC according to various ultrasonic extraction conditions, the relationship among the concentrations of C-PC, process time, and ultrasonic frequency was analyzed by a 3-D contour plot to find its optimal condition as an inflection point.

### 4.3. Structural Changes of C-PC in the Extracts from Two Different Extraction Processes

The patterns of change in the amounts of α,β-subunits of C-PC in two extracts were compared via high-performance liquid chromatography (HPLC, Dionex ultimate 3000, Dionex Co., Sunnyvale, CA, USA) equipped with a 3000 pump (LPG-3400SD, Dionex Co., USA) and an injector (WPS-3000SL ANAYTICAL, Dionex Co., ID × L, 0.18 × 550 mm Viper 550 mm, USA) with a C5 column (Discovery BIO Widepore, 250 × 4.6 mm, 5 μm, Supelco, Sigma, St. Louis, MO, USA) under the following conditions: the injection volume was 20 μL and the mobile phase was 20% (*v*/*v*) acetonitrile (ACN) mixed with 0.1% (*v*/*v*) trifluoroacetic acid (TFA) at 25 °C and a 1.0 mL/min flow rate under isocratic conditions for 45 min. The absorbance was measured with a UV detector at 580 and 640 nm and compared to the peaks of a standard C-PC (Sigma, St. Louis, MT, USA). For UV scanning of extracts and the C-PC standard, a photo diode array (PDA) spectrum was collected using a UV spectrophotometer (DAD-3000, Dionex Co.) to compare the α- and β-subunits of C-PC in the extracts [13,14].

### 4.4. Cell Cytotoxicity of the Extracts from Two Different Extraction Processes

The cytotoxicity of extracts (Control, WE and UE) against mouse macrophage cells (Raw264.7, ATCC, Manassas, VA, USA) was measured using the 3-(4,5-dimethythiazo-2-yl)-2,5-dipheny-tetrazoliumbromide (MTT) method [22]: first, 1.0 × 10^6^ cells/well of human macrophage cells were cultivated in a 96-well plate for 24 h. The extracts were added to each well at different concentrations (0.25, 0.50, 0.75, and 1.0 mg/mL) and further grown in a CO_2_ incubator for 24 h. Then, 5 μg/mL of MTT solution was added to each well, and the supernatant was removed after 4 h of cultivation. Then, 10 μL of acid-isopropanol (0.04 N HCl in isopropanol) was added to each well, and the absorbance was measured at 565 nm in a microplate reader (M1000 PRO; Infinite, Mannedorf, Switzerland). Cell cytotoxicity was calculated as the ratio of control cell growth (no sample) and growth in treated samples.

### 4.5. Measurement of Anti-Inflammatory Activities of Two Different Extracts

To estimate the anti-inflammation activities of the extracts, the production of nitric oxide (NO) from the growth of mouse macrophages (RAW264.7) was measured using a modified Green assay [23]: 1.0 × 10^6^ cells/mL were placed into a 96-well plate with RPMI 1640 medium containing 100 U/mL of penicillin and 100 μg/mL of streptomycin (85,886; Sigma), enriched with 10% (*v*/*v*) fetal bovine serum (FBS, GIBCO, Carlsbad, CA, USA). The cells were cultivated with different concentrations of samples (0.25, 0.50, 0.75, and 1.0 mg/mL) for 2 h. Afterwards, 1 μg/mL lipopolysaccharides (LPS) were added to this mixture, which was further cultivated in 5% CO_2_ in an incubator at 37 °C for 24 h. During the cell culture, the NO generated from the cell was measured and used as a control. Resveratrol was used as a positive control, and C-PC was used as a standard. Then, 50 μL of the supernatant from each well in a 96-well plate was collected using a centrifugal separator (Combi 514R; Hanil Science Medical, Daejeon, Korea) and allowed to react with 50 μL of Griess reagent for 5 min before measuring the absorbance of the mixture at 540 nm using a microplate reader with NaNO_2_ as a standard.

### 4.6. Determination of Antioxidant Activities of the Extracts from Two Different Processes

To measure the antioxidant activities of the extracts, α,α-diphenyl-β-picrylhydrazyl (DPPH) free radical scavenging activity was employed using the Dietz method [24]: after 150 μL of 0.1 mM DPPH solution prepared with methanol solvent was mixed with 150 μL of extract, this mixture was wrapped in foil and left unattended for 30 min at room temperature in the dark. The optical density was measured at 517 nm using a microplate reader (Thermo Fisher Scientific, Waltham, MA, USA). The relevant values were estimated as DPPH radical scavenging activity (%).

### 4.7. Statistical Analysis

All experiments were performed at least three times. The means of data and significant differences among samples were evaluated by ANOVA (SAS Institute, Cary, NC, USA) (*p* < 0.05).

## 5. Conclusions

Complex ultrasonic processes combined with other extraction systems have been considered to be effective for breaking rigid cell walls at a relatively low process temperature. However, a single ultrasonic extraction is not often employed to obtain bioactive substances from natural resources, due to the difficulties of controlling frequency with input power in an ultrasonicator and the limitations in the scale-up process for industrial production. Therefore, this work proves that an optimized simple ultrasonic extraction process (UE) can greatly increase the yields of three major pigments, C-PC, APC, and PE, from *Spirulina maxima*. Yields were even higher than those from other extraction processes that usually combine various pretreatments and extraction solvents. This work also showed that the amounts of α- and β-subunits of C-PC, due to a sharp decrease in C-PC yields under harsh conditions, greatly varied in HPLC chromatograms and UV spectra according to extraction conditions. More specifically, the HPLC peak patterns of α- and β-subunits of C-PC in the UE were very similar to those of an intact C-PC in a standard form, while the extract from water extraction at room temperature for 24 h (WE) showed a large reduction in the amount of α-subunits of C-PC, but not β-subunits. The UE also showed better antioxidant and anti-inflammatory activities than those of the WE. These results lead to the hypothesis that structural changes in C-PC influence biological effects as well as extraction yields, specifically because the biologically active part of C-PC, based on HPLC chromatograms and UV scanning, is the α-subunit and not the β-subunits. Therefore, these results show that a simple but efficient ultrasonic extraction method can be employed to obtain fairly high amounts of intact forms of very heat-sensitive C-PC, which can result in a reduction of production costs and can promote the use of single C-PC components rather than the consumption of entire powders with unpleasant tastes. These results also imply that the increase in biological effects of ultrasonic extracts could be attributed to an increase in the yields of intact C-PC and other biologically active substances in *Spirulina*. Further comprehensive studies should be carried out to reveal, in detail, the effects of each component in the extracts on its biological activities. 

## Figures and Tables

**Table 1:** Phycobiliprotein content in *S. maxima* samples derived via different extraction processes and measured with a spectrophotometer.

Sample	Temperature (°C)	Frequency (kHz)	Extraction Time (h)	C-Phycocyanin (mg/mL)	Allophycocyanin (mg/mL)	Phycoerythrin (mg/mL)
Control ^1^	4	-	24	9.8 ± 0.02	2.3 ± 0.02	0.7 ± 0.01
WE ^2^	25	-	24	5.7 ± 0.01	1.2 ± 0.05	0.3 ± 0.01
UE ^3^	25	60	1	8.6 ± 0.04	2.3 ± 0.03	0.5 ± 0.01
			2	9.2 ± 0.13	2.5 ± 0.06	0.7 ± 0.01
			3	11.3 ± 0.06 *	3.1 ± 0.02 *	0.8 ± 0.01 *
			4	10.9 ± 0.34 *	3.0 ± 0.02 *	0.5 ± 0.01
			5	9.9 ± 0.07	2.7 ± 0.01	0.6 ± 0.01
		20	3	10.2 ± 0.04	2.9 ± 0.03	0.4 ± 0.01
		40		11.3 ± 0.13 *	3.1 ± 0.05 *	0.8 ± 0.01 *
		60		10.9 ± 0.08	3.0 ± 0.07 *	0.6 ± 0.01
		80		11.2 ± 0.16 *	2.7 ± 0.03	0.4 ± 0.01
		100		10.8 ± 0.14 *	3.0 ± 0.08 *	0.7 ± 0.01

^1^ Control: *S. maxima* extract with water at 4 °C for 24 h. ^2^ WE: *S. maxima* extract with water at 25 °C for 24 h. ^3^ UE: *S. maxima* extract with water at 25 °C for 3–5 h by ultrasonification. * Significant difference, *p* < 0.05.

**Table 2:** Cytotoxicity of *Spirulina maxima* extracts in macrophage (RAW 264.7) cells.

Sample	Treatment Concentration (mg of *S. maxima* Extracts Powder/mL)			
	0.25	0.50	0.75	1.00
WE ^1^	1.58 ± 0.009	6.21 ± 0.101	11.89 ± 1.930	14.06 ± 1.027 *
UE ^2^	2.01 ± 0.057	7.74 ± 0.160	12.52 ± 1.021 *	15.48 ± 0.994 *

^1^ WE: *S. maxima* water extraction at 25 °C for 24 h. ^2^ UE: *S. maxima* extract by ultrasonication. * Significant difference, *p* < 0.05.

## Abbreviations

C-PC	C-phycocyanin
APC	allophycocyanin
PE	phycoerythrin
UE	ultrasonic extraction
WE	water extraction
